# Supplementary material for: Tailored PGE2 Immunomodulation of moDCs by Nano-Encapsulated EP2/EP4 Antagonists
Source: Int J Mol Sci. 2023 Jan 11;24(2):1392. doi: 10.3390/ijms24021392 (PMC9866164; doi:10.3390/ijms24021392)
Supplement: Supplementary file 1 [file ijms-24-01392-s001.zip › Sup Figure S1.pdf]

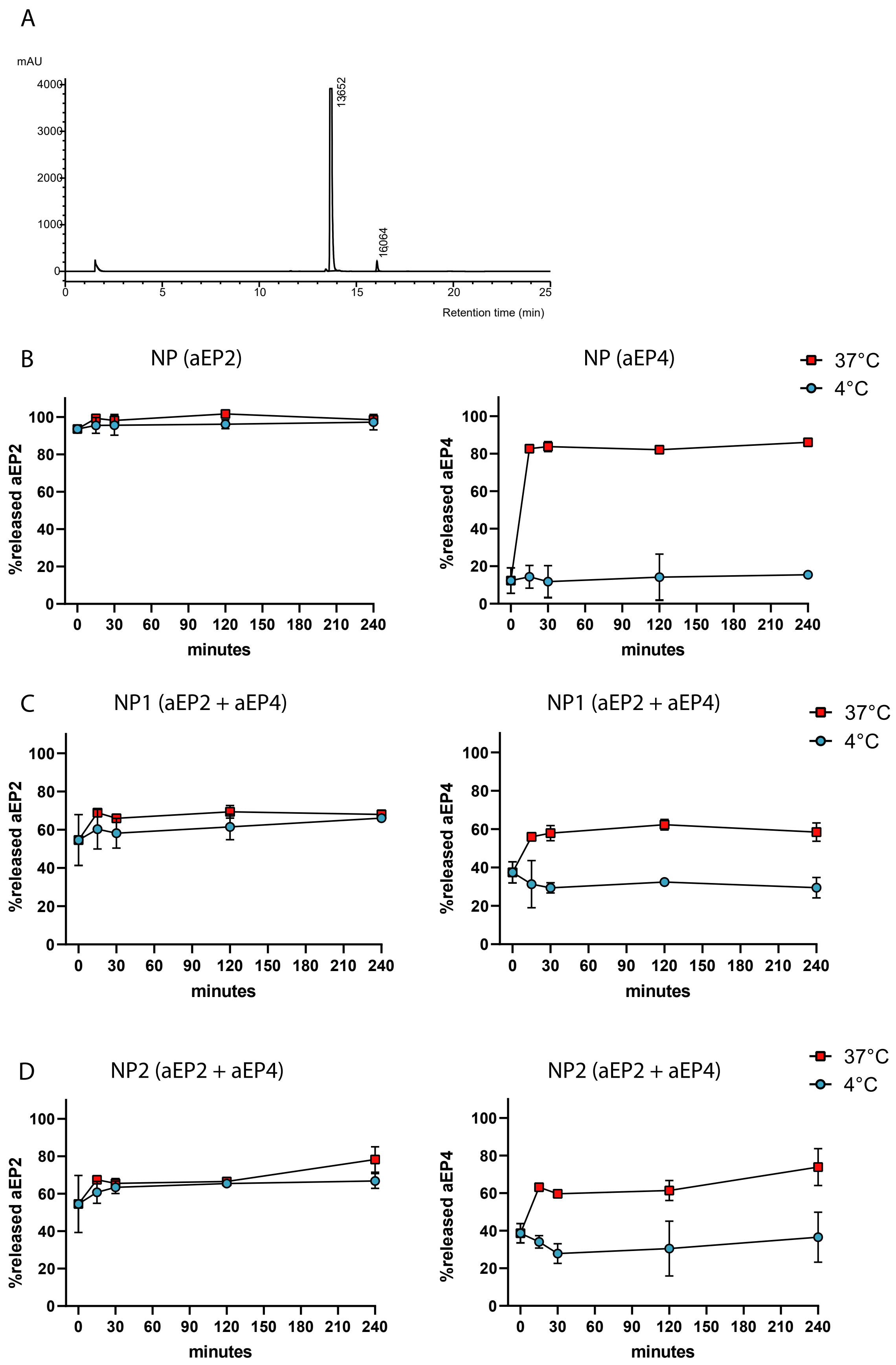

Figure S1: Nanoparticle characterization. (A) HPLC detection of aEP2 and aEP4. Percentage of released aEP2 and aEP4 from (B) NP (aEP2) NP (aEP4), (C) NP1 (aEP2 + aEP4), and (D) NP2 (aEP2 + aEP4) over 240 minutes at 37 °C (red) or 4 °C (blue). Each value represents mean ± SD of n ≥ 2 NP batches.

Sup Figure S1
