# Supplementary material for: Tailored PGE2 Immunomodulation of moDCs by Nano-Encapsulated EP2/EP4 Antagonists
Source: Int J Mol Sci. 2023 Jan 11;24(2):1392. doi: 10.3390/ijms24021392 (PMC9866164; doi:10.3390/ijms24021392)
Supplement: Supplementary file 1 [file ijms-24-01392-s001.zip › Sup Figure S2.pdf]

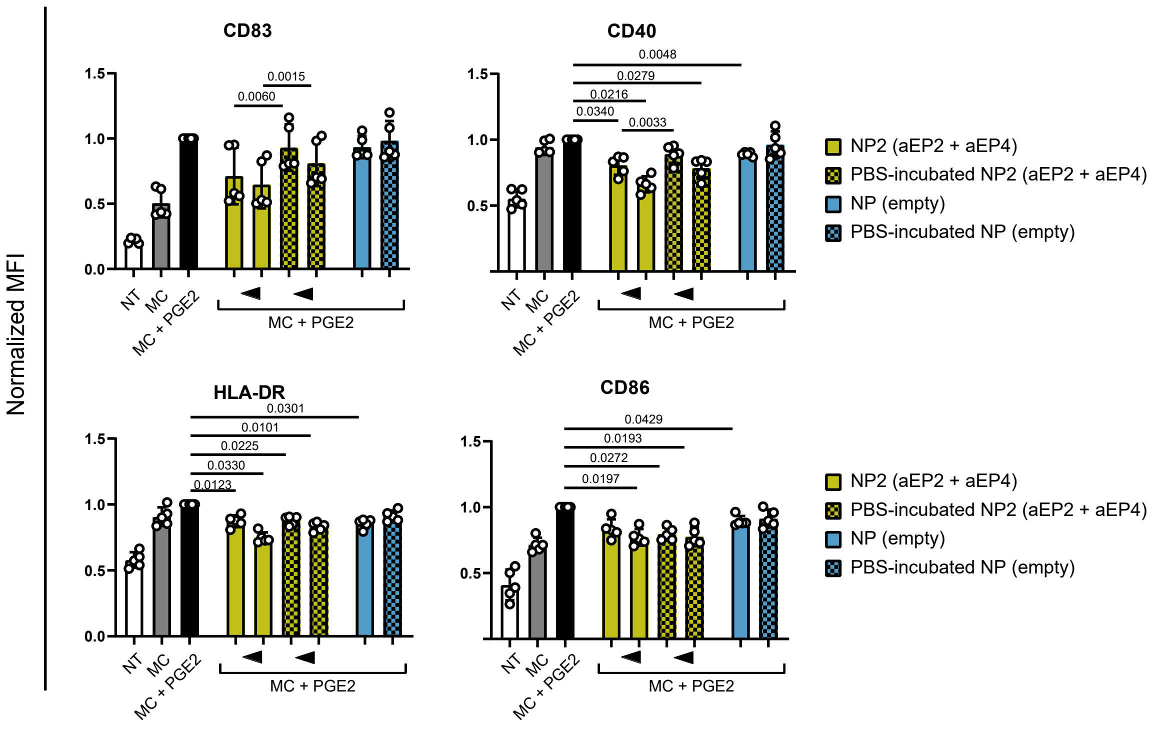

Figure S2: Normalized expression of CD83, CD40, HLA-DR, and CD86 on moDCs after 24 h of stimulation with PBS-incubated or non-incubated NPs. Control moDCs were left NT or stimulated with MC either in the presence or absence of PGE2. MoDCs were stimulated with MC + PGE2 with either PBS-incubated or non-incubated NPs at low (30 µM aEP2) and high (60 µM aEP2) concentrations (indicated by arrow). PBS-incubated and or non-incubated NPs (empty) were used as control which matched the highest NP concentration. The expression was normalized to the MC + PGE2 control moDCs. Each dot represents one donor and the bar is the mean ± SD for n=5. P values were calculated on non-normalized data with one-way ANOVA with Tukey multiple comparison correction.

Sup Figure S2
