# Supplementary material for: Tailored PGE2 Immunomodulation of moDCs by Nano-Encapsulated EP2/EP4 Antagonists
Source: Int J Mol Sci. 2023 Jan 11;24(2):1392. doi: 10.3390/ijms24021392 (PMC9866164; doi:10.3390/ijms24021392)
Supplement: Supplementary file 1 [file ijms-24-01392-s001.zip › Sup Figure S3.pdf]

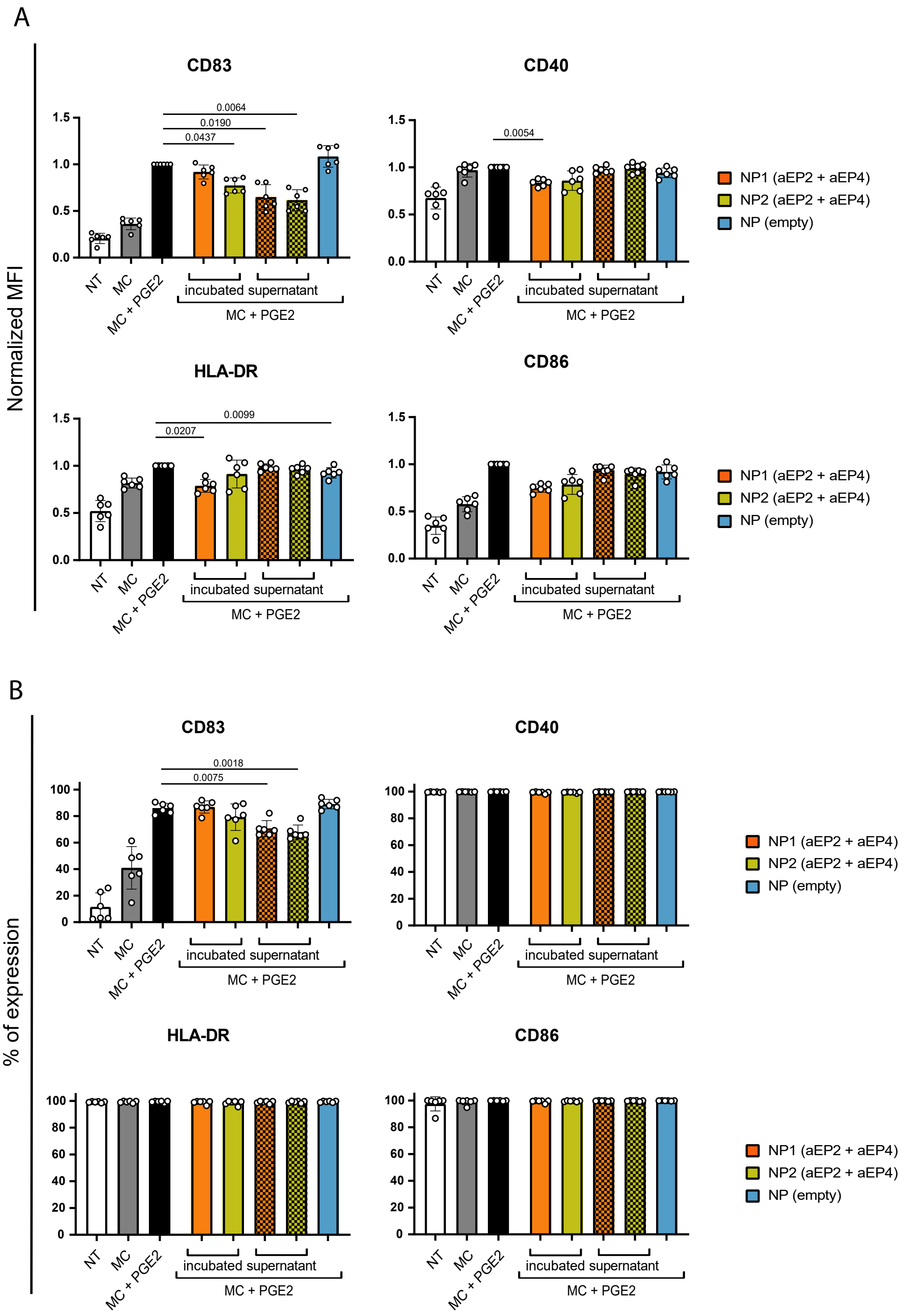

Figure S3: Co-stimulatory marker expression on moDCs after 24 h of stimulation with PBS-incubated NPs or supernatant of PBS-incubated NPs. (A) Normalized expression of CD83, CD40, HLA-DR, and CD86 and (B) percentage of CD83, CD40, HLA-DR, and CD86 expressing moDCs. Control moDCs were left NT or stimulated with MC either in the presence or absence of PGE2. MoDCs were stimulated with MC + PGE2 and either PBS-incubated NPs or the supernatant of incubated NPs at a matched 30 µM aEP2 concentration. Empty NPs were used as controls to match the highest NP concentration. Each dot represents one donor and the bar is the mean ±SD for n=5. P values were calculated on non-normalized data with one-way ANOVA with Tukey multiple comparison correction.

Sup Figure S3
