# Supplementary material for: Tailored PGE2 Immunomodulation of moDCs by Nano-Encapsulated EP2/EP4 Antagonists
Source: Int J Mol Sci. 2023 Jan 11;24(2):1392. doi: 10.3390/ijms24021392 (PMC9866164; doi:10.3390/ijms24021392)
Supplement: Supplementary file 1 [file ijms-24-01392-s001.zip › Sup Figure S4.pdf]

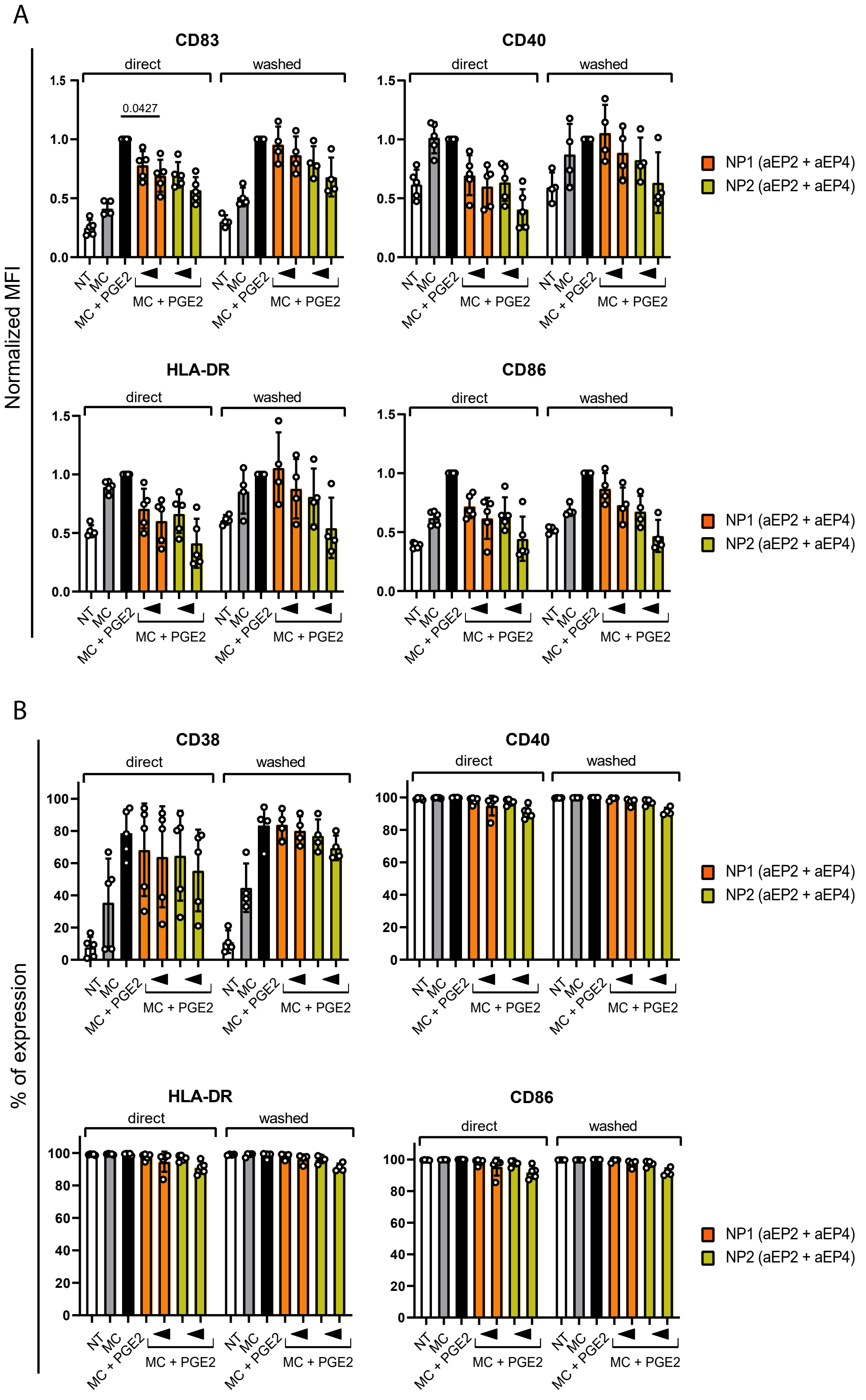

Figure S4: Co-stimulatory marker expression on moDCs 24 h after the addition of NPs, either directly or washed cells. (A) Normalized expression of CD83, CD40, HLA-DR, and CD86 and (B) percentage of CD83, CD40, HLA-DR, and CD86 expressing moDCs. Controls were left NT, stimulated with MC and MC with PGE2. MoDCs were stimulated with NPs directly or the cells were washed after one hour of stimulation. Two batches of NPs were used with a matched low (30 µM) and high (60 µM) aEP2 concentration (indicated by arrow). The expression was normalized to the MC + PGE2 control Each dot represents one donor and the bar is the mean ±SD with n=5 for direct and n=4 for washed conditions. P values were calculated on non-normalized data with one-way ANOVA with Tukey multiple comparison correction.

Sup Figure S4
