# Supplementary material for: Tailored PGE2 Immunomodulation of moDCs by Nano-Encapsulated EP2/EP4 Antagonists
Source: Int J Mol Sci. 2023 Jan 11;24(2):1392. doi: 10.3390/ijms24021392 (PMC9866164; doi:10.3390/ijms24021392)
Supplement: Supplementary file 1 [file ijms-24-01392-s001.zip › Sup Figure S5.pdf]

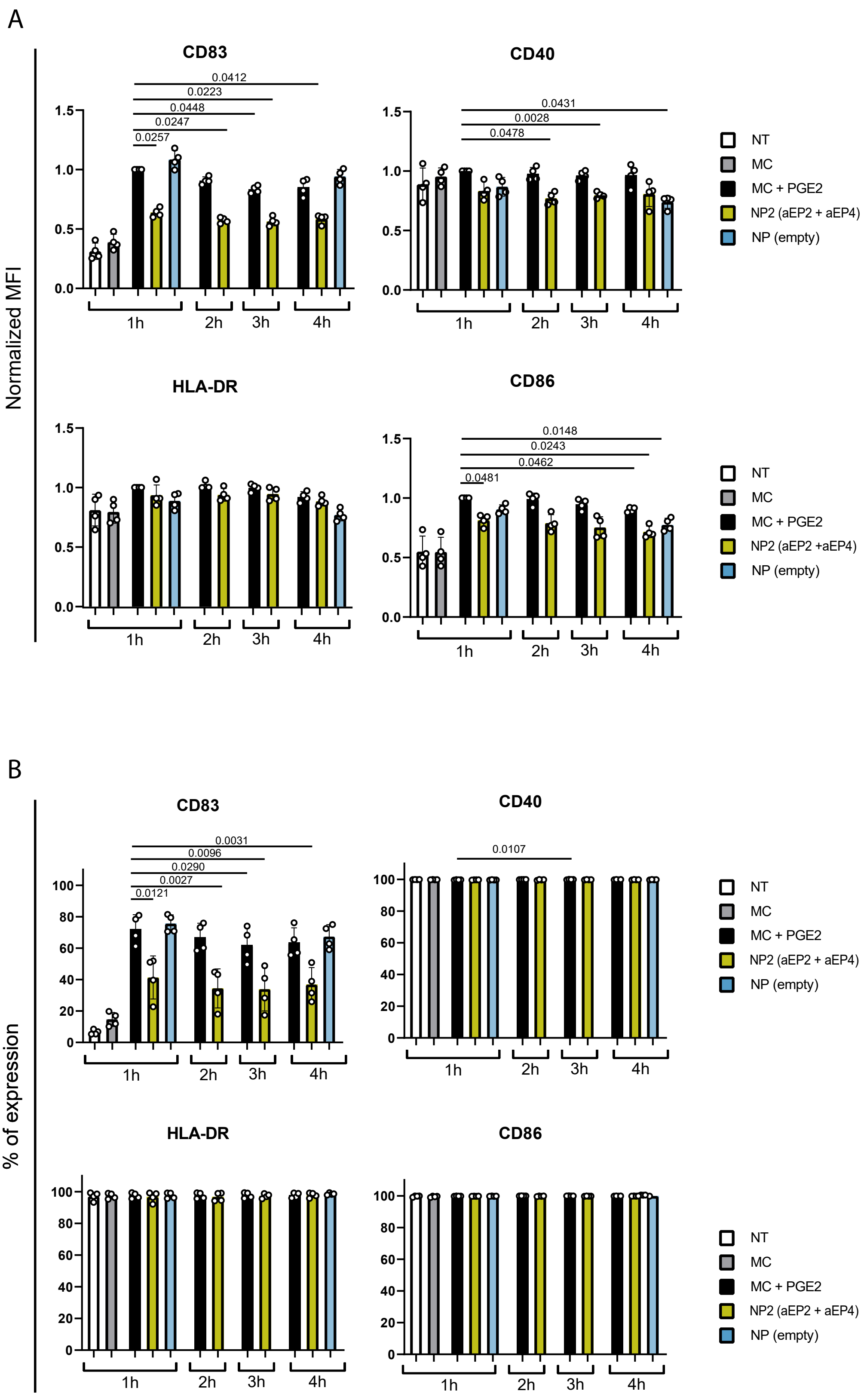

Figure S5: Co-stimulatory marker expression on moDCs after 24h of stimulation with NPs for varied hours. (A) Normalized expression of CD83, CD40, HLA-DR, and CD86 and (B) percentage of CD83, CD40, HLA-DR, and CD86 expressing moDCs. Control moDCs were left NT, stimulated with MC and MC with PGE2. MoDCs were stimulated with NPs and the MC + PGE2 was given either after 1-, 2-, 3-, or 4-hours. Empty NPs matched to the highest NP concentration were used as a control for 1 h and 4 h of stimulation. The control with MC + PGE2 was repeated for each timepoint. The expression was normalized to the MC + PGE2 expression after 1 h of stimulation. Each dot represents one donor and the bar is the mean ±SD n=4. P values were calculated on non-normalized data with one-way ANOVA with Tukey multiple comparison correction.

Sup Figure S5
