# Supplementary material for: Tailored PGE2 Immunomodulation of moDCs by Nano-Encapsulated EP2/EP4 Antagonists
Source: Int J Mol Sci. 2023 Jan 11;24(2):1392. doi: 10.3390/ijms24021392 (PMC9866164; doi:10.3390/ijms24021392)
Supplement: Supplementary file 1 [file ijms-24-01392-s001.zip › Sup Figure S6.pdf]

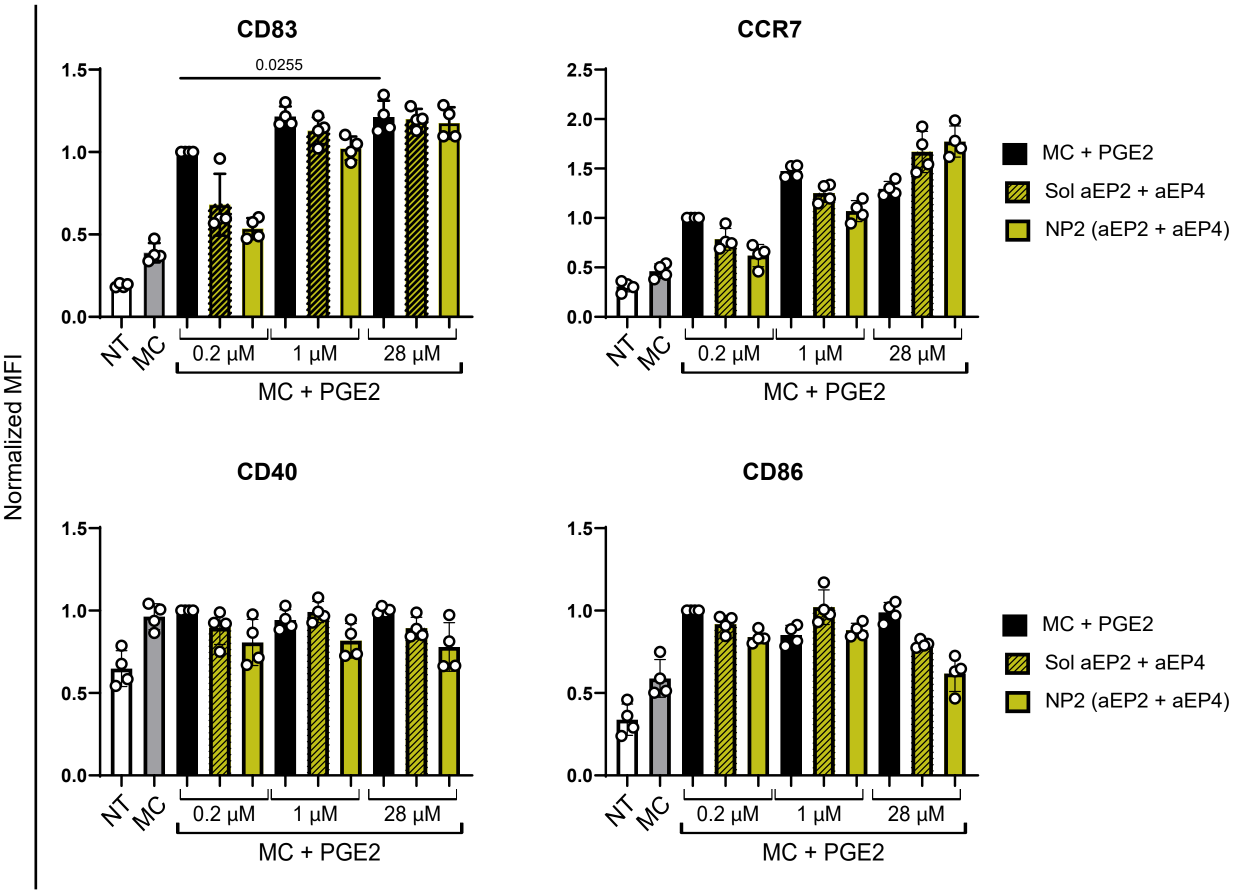

Figure S6: Normalized expression of CD83, CCR7, CD40, and CD86 on moDCs after 24 h of stimulation with encapsulated or soluble aEP2 + aEP4 and increasing PGE2 concentration. Control moDCs were left NT, stimulated with MC and MC with three different concentrations of PGE2: 0.2, 1, or 28 µM. MoDCs were stimulated with encapsulated or soluble aEP2 + aEP4 and MC + PGE2 with the three different PGE2 concentrations. The encapsulated or soluble antagonists concentration matched a final concentration of 60 µM aEP2 and 1.25 µM aEP4. The expression was normalized to the MC + 0.2 µM PGE2 control. Each dot represents one donor and the bar is the mean ± SD for n=4. P values were calculated on non-normalized data with one-way ANOVA with Tukey multiple comparison correction.

Sup Figure S6
